# Fingerprinting PCR Reveals Potential Dissemination of Multidrug Efflux System Genes and Antimicrobial Resistance in *Staphylococcus aureus* Across Primary Healthcare Units in Brazil

**DOI:** 10.1155/ijm/9287240

**Published:** 2026-05-14

**Authors:** Ilderlane da Silva Lopes, Ana Júlia Silva Moreira, Mariana de Barros, Leonardo Moises Sales Bueno, Giarlã Cunha da Silva, Jéssica Nogueira Rosa, Jéssica Lobo Albuquerque Caldeira, Rodrigo Alves Barros, Denise Mara Soares Bazzolli, Maria Aparecida Scatamburlo Moreira

**Affiliations:** ^1^ Department of Veterinary, Laboratory of Bacterial Diseases, Universidade Federal de Viçosa, Viçosa, Minas Gerais, Brazil, ufv.br; ^2^ Department of Microbiology, Bacteria Molecular Genetics Laboratory, Universidade Federal de Viçosa, Viçosa, Minas Gerais, Brazil, ufv.br

**Keywords:** antimicrobials, dogs, humans, One Health, public health system, resistance mechanism

## Abstract

Multidrug efflux systems (MESs) are major contributors to antimicrobial resistance (AMR) in *Staphylococcus aureus*, yet their role in primary healthcare settings is poorly understood. Under a One Health framework, we investigated MES‐mediated resistance in 38 *S. aureus* isolates (27 from humans, 11 from dogs) from three Basic Health Units (BHUs) in Viçosa, Brazil. Isolates were characterized by antimicrobial susceptibility testing, PCR for six key efflux genes, and (GTG)_5_‐PCR fingerprinting. Phenotypic efflux activity was evaluated using ethidium bromide fluorescence assays. Thirty‐seven isolates were resistant to at least one antimicrobial, most commonly penicillin (57.9%) and erythromycin (55.3%), while all remained susceptible to chloramphenicol, trimethoprim, and linezolid. While the *msrA* gene was rare (10.5%), other efflux genes like *norA/B/C*, *lmrS*, and *tet38* were nearly ubiquitous (> 94%). This high genetic prevalence contrasted with low phenotypic resistance, indicating that most MES genes were not expressed. Fingerprinting revealed seven genetic clusters, demonstrating the circulation of closely related strains between human and animal hosts across different health units. Eight isolates showed clear genotype–phenotype concordance, with MES activity confirmed phenotypically. The four *msrA*‐positive, erythromycin‐intermediate isolates formed two clonal groups (100% similarity): one shared between two users from different BHUs and another shared between a healthcare worker and a dog from different BHUs, providing direct evidence of interhost and cross‐geographic AMR dissemination. Moreover, co‐colonization of a single individual with two genetically distinct tetracycline‐resistant strains (60% similarity) suggests possible horizontal gene transfer. Although phenotypic MES‐mediated resistance was limited (21%), we demonstrate the potential AMR spread across hosts and geographic boundaries, as primary healthcare settings harbor a significant reservoir of MES genes in *S. aureus* even if they are silent. These results highlight the critical need for integrated One Health surveillance in community settings to mitigate AMR dissemination.

## 1. Introduction

The Brazilian Unified Health System (SUS) is one of the world’s largest public healthcare networks, providing universal access to services ranging from primary care to complex procedures. Within SUS, Basic Health Units (BHUs) serve as the frontline for community health, offering preventive care and early disease management [1, 2]. Despite their critical role, BHUs remain understudied as potential reservoirs of antimicrobial resistance (AMR), a global threat exacerbated by the misuse of antibiotics and bacterial adaptation mechanisms [3].

Among these mechanisms, multidrug efflux systems (MESs) enable bacteria to expel diverse antimicrobials, reducing intracellular drug concentrations and conferring resistance [4]. Studies confirm that bacteria can use efflux systems as defense mechanisms to expel toxic compounds and for self‐protection against environmental pollutants, including antimicrobials [5, 6]. MES are classified into families, such as the ATP‐binding cassette (ABC) superfamily, the multidrug and toxic compound extrusion (MATE) family, the major facilitator superfamily (MFS), and the small multidrug resistance (SMR) family, based on structure and energy coupling [6, 7]. While some MES are drug‐specific, others confer broad‐spectrum resistance [8].

In *Staphylococcus aureus*, a major human and animal pathogen, MES are represented by the MFS, SMR, MATE, and ABC families, with the MFS family being the most extensively studied in staphylococci [9]. Although MES are well‐characterized in hospital‐associated *S. aureus*, their prevalence and dissemination in primary care settings remain poorly understood. This lack of knowledge is concerning because antibiotic use in these settings is often empiric and unmonitored, as prescriptions are often made without a prior antibiogram assay. Moreover, the One Health perspective further highlights the urgency of this issue. Close contact between humans and animals in community settings, such as stray dogs (unconfined animals that free‐roam within the community) found wandering in and around the BHUs premises, may facilitate cross‐species AMR transmission [10]. Yet, most studies focus on hospitals, neglecting the potential role of BHUs as silent community reservoirs of AMR.

Thus, this study investigates the potential spread of MES‐mediated resistance in *S. aureus* within BHUs of Viçosa, Brazil. We hypothesized that BHUs may function as silent community reservoirs of efflux‐associated AMR, allowing the circulation of resistance genes between humans and animals. Hence, we aimed to (i) characterize the AMR profiles and the prevalence of key MES genes in isolates obtained from humans (patients and healthcare workers) and stray dogs; (ii) assess the genetic relatedness of isolates across hosts and sampling periods to identify potential dissemination patterns within and between BHUs; and (iii) evaluate phenotypic efflux activity to correlate genotypic profiles with observed resistance phenotypes. By integrating phenotypic, molecular, and epidemiological approaches, this work addresses a critical gap in understanding AMR dynamics in primary healthcare settings and underscores the need for integrated One Health surveillance to mitigate resistance spread in the community.

## 2. Materials and Methods

### 2.1. Isolation and identification of *Staphylococcus aureus*

Bacterial isolates were obtained from three BHUs in Viçosa, Minas Gerais, Brazil, designated W, Y, and Z. Sampling occurred during two distinct periods: the dry season (March–April) and the rainy season (August–September) of 2019. A total of 193 human participants (healthcare professionals and patients) and 107 free‐roaming (stray) dogs were sampled. For humans, swabs were collected from the dominant hand—at the end of the work shift for healthcare professionals and upon arrival at the BHUs for patients—using sterile swabs in Stuart transport medium (Absorve, CRAL). The swabbing procedure involved moving from the lower palm to the fingertips and back to the wrist in three circular motions. Dog samples were collected with the same swab type, gently swiping in circular motions across the external surface of the nose.

The swabs were streaked in duplicate on Petri dishes containing 5% sheep blood agar and incubated aerobically at 37°C for 24–48 h. Following pure culture isolation, the bacteria were identified using classical microbiology tests, General Chromogenic Agar (Probac), and confirmed as *S. aureus* by PCR targeting the *nuc* gene (nuc‐f: 5′‐GCG​ATT​GAT​GGT​GAT​ACG​GTT‐3′; nuc‐r: 5′‐AGC​CAA​GCC​TTG​ACG​AAC​TAA​AGC‐3′),according to amplification protocols described by Yang et al. [11].

In total, 107 dogs, 82 healthcare professionals, and 111 patients were included in the study across the three BHUs.

### 2.2. Antimicrobial Susceptibility Testing

Antimicrobial susceptibility testing was performed using primary antimicrobials commonly prescribed in human and veterinary medicine, using the Kirby–Bauer disk diffusion method. The *S. aureus* isolates were reactivated in brain heart infusion (BHI) broth and incubated at 37°C until reaching a turbidity equivalent to 0.5 on the McFarland scale, measured using a spectrophotometer (BioMate 3 Series Spectrophotometer—Thermo Electron Corporation).

The inocula were then spread with a sterile swab onto Mueller–Hinton (MH) agar plates in duplicate, and antibiotic disks (Sensidisc) were applied: penicillin G (10 IU), chloramphenicol (30 μg), ciprofloxacin (5 μg), doxycycline (30 μg), erythromycin (15 μg), gentamicin (10 μg), tetracycline (30 μg), nitrofurantoin (300 μg), norfloxacin (10 μg), sulfonamides (300 μg), sulfamethoxazole/trimethoprim (25 μg), linezolid (30 μg), and trimethoprim (5 μg). Plates were incubated at 37°C for 24 h, and the inhibition zones were interpreted according to the guidelines provided by the Clinical and Laboratory Standards Institute [12].

### 2.3. Detection of MES Genes

The detection of MES genes was performed using the polymerase chain reaction (PCR) technique. DNA extraction from the *S. aureus* isolates was carried out using the Wizard Genomic DNA Purification Kit (PROMEGA). Amplification reactions were prepared in a total volume of 25 μL, consisting of 12.5 μL of GoTaq Green Master Mix (Promega), 20 ρmol of each primer, 2 μL of genetic material (50 ng), and 6.5 μL of nuclease‐free water. The selection of MES genes targeted in this study was based on their documented involvement in AMR and colonization potential in *S. aureus* [13–19]. Table S2 lists the genes analyzed and the primers used [20].

For the *norA*, *norC*, *lmrS*, and *tet38*​ genereactions, the control was the *S. aureus* isolate 4c, a previously sequenced laboratory strain [21]. For the *norB* and *msrA* genes, the size of the amplicon formed was used as the control.

### 2.4. Molecular Epidemiology

To investigate the dissemination of MES genes of *S. aureus* among humans from the BHUs and stray dogs, PCR methodology based on repetitive sequences (rep‐PCR) was used, specifically the (GTG)_5_‐PCR technique. This technique was selected for its demonstrated efficacy in generating strain‐specific genomic fingerprints suitable for cluster analysis and preliminary tracking of bacterial dissemination in epidemiological studies [22–24]. The primer (GTG) 5′‐GAG​GGT​GGC​GGT​TCT‐3′ was used as described by Lieckfeldt et al. [25]. The PCR mixture was prepared with a final volume of 25 μL, containing 5 μL of 5*x* Green Buffer (GoTaq Flexi Buffer), 0.2 mM of dNTPs, 1 μM of universal primer (GTG)_5_, 1.5 mM of MgCl2, and 2 U of GoTaq Flexi DNA polymerase (Promega). The DNA used in the reaction was standardized to 25 ng. The PCR conditions were as follows: initial denaturation at 95°C for 5 min, followed by 30 cycles of denaturation at 94°C for 1 min, annealing at 40°C for 1 min, and extension at 78°C for 8 min. The final cycle was followed by a single extension step at 72°C for 5 min. The reactions were carried out in a BioRad‐C1000 TM Thermal Cycler, and the products were assessed using agarose gel electrophoresis at 1.8%.

For the interpretation of the results, a matrix was constructed for the amplified fragments coded in a binary system, assigning a value of (1) for the presence and (0) for the absence of bands in the virtual gels. From this matrix, a dendrogram was generated using the statistical software PAST v. 3.11 [26] with the unweighted pair group method with arithmetic mean (UPGMA) clustering method and the Jaccard index coefficient. Data analysis was performed by adopting a similarity threshold of 80%, as used by Lagha et al. [27].

### 2.5. Phenotypic Detection of MES Activity

To investigate AMR prevalence and the role of MESs, phenotypic assays were associated with molecular analyses on *S. aureus* isolates obtained from human and canine sources at BHUs in Viçosa, Minas Gerais, Brazil.

#### 2.5.1. Selection of Isolates

Only *S. aureus* isolates carrying MES‐related genes and exhibiting a matching resistant or intermediate phenotype to the corresponding antimicrobial were included in subsequent analyses.

#### 2.5.2. Minimum Inhibitory Concentration (MIC)

The MIC of antimicrobials was evaluated both in the absence and presence of carbonyl cyanide *m*‐chlorophenylhydrazone (CCCP), an efflux system inhibitor, at a concentration of 25 μg/mL. MIC determination was performed using a 96‐well microtiter plate with MH broth following CLSI guidelines [12] and employing a final inoculum of 5 × 10^5^ CFU/mL. The test was conducted in duplicate and repeated twice, with the MIC defined as the lowest antimicrobial concentration that inhibited bacterial growth. Results were compared with the control strain (*S. aureus* ATCC 25923).

#### 2.5.3. Quantification of Efflux Activity

The protocol by Kaatz et al. [28] was followed with modifications by Barros et al. [20]. Ethidium bromide (EtBr) was used as a probe since it emits weak fluorescence in aqueous solution and becomes highly fluorescent in nonpolar and hydrophobic environments, especially when it penetrates the bacterial cell wall and accumulates in the cytoplasm [29]. *S. aureus* isolates were cultured overnight in BHI broth. The inoculum was diluted to an OD660 of 0.7–0.8 and rested at room temperature for 20 min. The OD_660_ was then adjusted to 0.4 with sterile BHI broth containing CCCP (100 μM) and EtBr (10 μg/mL). The cultures were rested for an additional hour at room temperature to allow the entry of EtBr into the bacteria. After incubation, 400 μL of the culture were centrifuged (4000 rpm/5 min), the supernatant discarded, and the pellet resuspended in 400 μL of phosphate‐buffered saline (PBS) at pH 7.2. Next, 50 μL of the suspension was distributed (in duplicates) into 500 μL microtubes that had previously received 50 μL of PBS, 50 μL of BHI with CCCP, and 50 μL of glucose (1 M). Fluorescence was monitored through 60 cycles of 10 s each in a Rotor‐Gene Q thermocycler (Qiagen) using an excitation wavelength of 530 nm and detection at 630 nm.

The fluorescence readings for each treatment were normalized by subtracting the mean fluorescence of the glucose treatment from the mean fluorescence of the PBS and CCCP treatments for each time point. The glucose treatment served as a positive control for efflux activity, as glucose acts as an energy source for the efflux pumps, leading to a maximal expulsion of EtBr and, consequently, the lowest possible fluorescence readings. These normalized trend curves were then plotted for visualization and interpretation. Efflux activity was confirmed when the fluorescence curve for the CCCP treatment consistently remained above the fluorescence curve for the PBS treatment.

## 3. Results

### 3.1. Overall Resistance Profile

A total of 38 *S. aureus* isolates were analyzed, including 27 obtained from the hands of healthcare professionals and patients at BHUs and 11 from the snouts of stray dogs that frequent the areas surrounding these units. Antimicrobial susceptibility testing revealed that 37 isolates (97.4%) were resistant to at least one agent. Resistance was predominantly associated with penicillin (57.9%) and erythromycin (55.3%), whereas susceptibility to most other antimicrobial classes was largely preserved. Complete susceptibility (100%) was observed for six antimicrobial classes: phenicols (chloramphenicol), oxazolidinones (linezolid), nitrofurans (nitrofurantoin), fluoroquinolones (norfloxacin), diaminopyrimidines (trimethoprim), and sulfonamide combinations (sulfamethoxazole). Intermediate resistance occurred for sulfonamide (34.2%) and ciprofloxacin (2.6%), while resistance to tetracyclines (tetracycline, doxycycline) and aminoglycosides (gentamicin) remained low (< 8%) (Table 1). This heterogeneous profile indicates that resistance in this setting is dominated by beta‐lactams and macrolides. The detailed resistance profile of each isolate is presented in Figure S1.

**TABLE 1:** Antimicrobial resistance profile of *Staphylococcus aureus* isolates from dog nostrils and human hands (healthcare workers and patients) in primary healthcare units (UBS), Viçosa, MG, Brazil (2019).

	Resistant		Sensitive		Intermediate	
Antimicrobial	*n*	%	*n*	%	*n*	%
Penicillin	22	57.9	16	42.1	0	0.0
Erythromycin	21	55.3	12	31.6	5	13.2
Tetracycline	3	7.9	35	92.1	0	0.0
Sulfonamide	3	7.9	22	57.9	13	34.2
Doxycycline	1	2.6	36	94.7	1	2.6
Gentamicin	1	2.6	36	94.7	1	2.6
Chloramphenicol	0	0.0	38	100.0	0	0.0
Linezolid	0	0.0	38	100.0	0	0.0
Nitrofurantoin	0	0.0	38	100.0	0	0.0
Norfloxacin	0	0.0	38	100.0	0	0.0
Sulfamethoxazole	0	0.0	38	100.0	0	0.0
Trimethoprim	0	0.0	38	100.0	0	0.0
Ciprofloxacin	0	0.0	37	97.4	1	2.6

### 3.2. Prevalence of MES Genes

**FIGURE 1:**
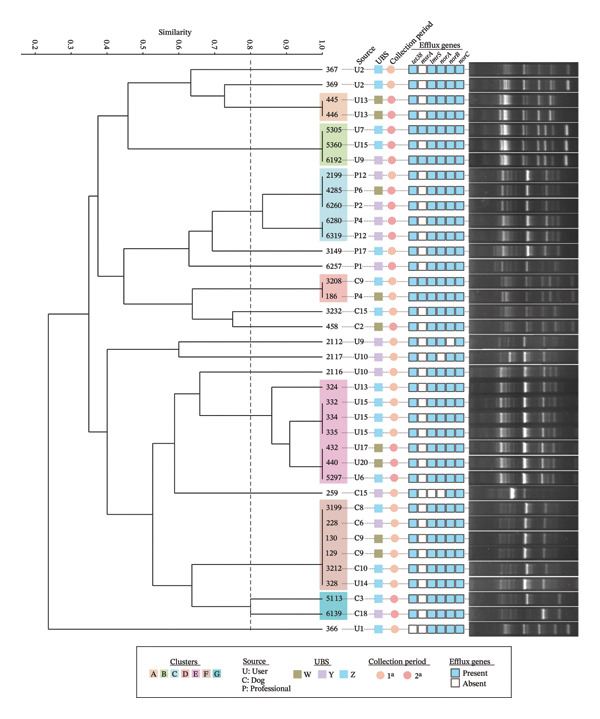
Dendrogram of 38 *Staphylococcus aureus* isolates from dogs and humans (patients and healthcare professionals) at three Basic Health Units (W, Y, and Z), Viçosa‐MG, 2019. The dendrogram was constructed with 80% similarity based on the clustering analysis of the digital fingerprint profiles (GTG)_5_‐PCR combined with multidrug efflux system genes. The origins of the isolates and the collection periods (1st: dry season and 2nd: rainy season) are indicated.

Among the MES genes analyzed, *norC* was present in all isolates (100%), followed by *tet38*, *lmrS*, and *norB* (97.4%) and *norA* (94.74%). The *msrA* gene, associated with erythromycin resistance [19], was the least frequent (10.53%). These results show a near‐ubiquitous presence of efflux pump genes, indicating a widespread genetic potential for resistance that contrasts with the more limited phenotypic resistance observed. Table S1 lists the genes analyzed and the primers used. The presence of these genes across isolates, grouped by origin and collection period, is illustrated in Figure 1.

### 3.3. Molecular Typing and Cluster Analysis

PCR fingerprinting with the (GTG)_5_ primer grouped the isolates into seven Clusters (A–G) based on genetic polymorphism profiles, while 11 isolates remained ungrouped (Figure 1). The clustering pattern showed a clear temporal structure, with Clusters A, B, and G containing only rainy‐season isolates, Clusters D and F containing only dry‐season isolates, and Clusters C and E spanning both seasons, indicating the persistence of some lineages year‐round.

Cluster A grouped two isolates with 100% shared polymorphic bands, originating from the same user. Clusters B and E highlighted the spread of isolates with high identity between users from different BHUs (Y–Z and W–Z, respectively), indicating dissemination between healthcare units. Cluster C grouped genetically identical isolates obtained from healthcare professionals working at BHUs W and Y across both seasons, suggesting sustained circulation within the professional environment. Cluster D contained two isolates with 100% genetic polymorphism identity from distinct hosts, a dog and a healthcare professional, located at different BHUs, providing evidence of cross‐species dissemination. This human–animal interaction is more clearly seen in Cluster F. This cluster included multiple isolates with identical fingerprints and a broad repertoire of MES genes, suggesting the circulation of these bacteria between different BHUs and probably carried by dogs that frequent nearby areas. This situation was also observed for cluster G, which included two highly similar isolates from dogs at different BHUs (Y–Z). Cluster E was the largest cluster, encompassing seven isolates and demonstrating dissemination of *S. aureus* between users from BHUs W and Z across distinct seasons. Taken together, the clustering patterns reveal interconnected transmission networks linking humans, animals, and healthcare units.

### 3.4. Genotype–Phenotype Association

**FIGURE 2:**
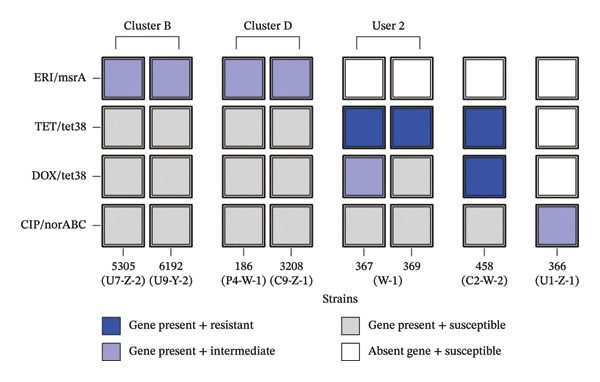
Matching between efflux‐related resistance genes and antimicrobial resistance phenotypes in eight selected *S. aureus* isolates. The heatmap displays the presence of resistance genes and corresponding phenotypic resistance to erythromycin (ERI), tetracycline (TET), doxycycline (DOX), and ciprofloxacin (CIP). Only isolates with concordant genotype–phenotype profiles were included. Strains are grouped according to epidemiological relationship. Color coding indicates genotype–phenotype combinations. Isolate codes are structured as follows: Host ID—BHU ID—Collection period, where U = user (patient), P = healthcare professional, C = canine isolate; BHU = Basic Health Unit W, Y, or Z; and period 1 or 2 indicates the sampling phase.

Eight selected isolates that demonstrated a clear link between a MES gene and its corresponding resistance phenotype (Figure 2). These isolates, sourced from users (*n* = 5), a professional (*n* = 1), and dogs (*n* = 2), included four erythromycin‐intermediate strains carrying the *msrA gene*, one ciprofloxacin‐intermediate strain with *nor* genes, and three tetracycline‐resistant strains with *tet38*. Some of these isolates (strains 5305/6192 and 186/3208) belonged to clusters with 100% fingerprint similarity and were recovered from different hosts, BHUs, or seasons, reinforcing the circulation of strains expressing efflux‐mediated resistance within the community. Moreover, strains 367 and 369, both tetracycline‐resistant, were isolated from the same person (User 2), suggesting either co‐colonization by distinct resistant strains or the local acquisition of resistance determinants.

### 3.5. Efflux Activity Assays

**FIGURE 3:**
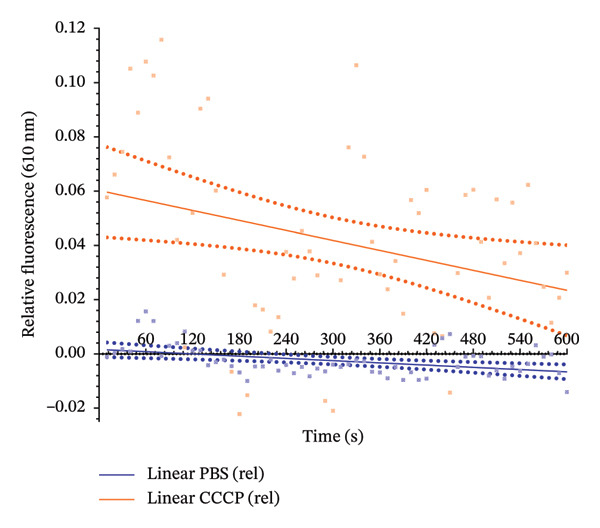
Ethidium bromide efflux activity curve for representative *Staphylococcus aureus* isolate 6192 from humans in Basic Health Units (UBS), Viçosa‐MG, Brazil. Blue squares show data points for the phosphate‐buffered saline (PBS) treatment, while orange squares represent the fluorescence in the presence of carbonyl cyanide *m*‐chlorophenylhydrazone (CCCP). The straight lines show the simple linear regression of each treatment, with dotted lines representing the 95% confidence interval. Multidrug efflux system activity is demonstrated by the CCCP trend line above the PBS line, indicating a higher accumulation of ethidium bromide due to efflux inhibition.

No reduction in MIC values was observed in the presence of the efflux pump inhibitor CCCP, suggesting the involvement of additional resistance mechanisms (data not shown). However, efflux activity was observed by the fluorescence assay with EtBr (Figure 3). The sustained higher fluorescence in the presence of CCCP indicates active efflux inhibition and intracellular EtBr accumulation, confirming functional efflux pump activity in these isolates.

## 4. Discussion

The AMR observed in *S. aureus* isolates highlights the ongoing challenges this pathogen poses in healthcare and community settings. While resistance to at least one antimicrobial was widespread, the sensitivity of all isolates to seven antimicrobials was unexpected given that the strains harbored numerous MES genes, indicating potential for broader resistance. This apparent contradiction suggests that strains circulating in these settings are genetically primed for resistance but remain phenotypically silent under the relatively low selective pressures typical of BHUs, in contrast to hospital environments where sustained antimicrobial exposure promotes rapid selection of resistant lineages [30, 31]. Such conditions may allow resistance determinants to persist without immediate phenotypic expression. These silent reservoirs may represent an underrecognized risk for the future emergence of clinically relevant resistance in community environments, underscoring the need for continuous surveillance even outside hospital settings.

Despite the low MDR profile, the widespread detection of MES genes remains epidemiologically relevant. Efflux systems are associated not only with AMR but also with biofilm formation, bacterial adhesion, quorum sensing, invasiveness [6], environmental persistence, and host adaptation [6, 32, 33], thereby enhancing bacterial fitness and virulence even in the absence of strong selective pressure. Moreover, these genes represent a reservoir for potential horizontal dissemination to other bacteria, facilitating the emergence of resistant pathogens within BHUs.

Therefore, molecular fingerprinting provided insights into bacterial spread. The (GTG)_5_‐PCR technique is efficient for taxonomic delineation of *Staphylococcus* spp. [24], with clusters comparable to PFGE [23], being a feasible and cost‐effective approach [22]. PCR fingerprinting grouped isolates into seven Clusters (A–G) with clear temporal structure. Clusters A, B, and G were restricted to the rainy season, Clusters D and F to the dry season, and Clusters C and E spanned both seasons, indicating year‐round persistence (Figure 1). This adaptability to diverse environmental conditions [34] ensures *S. aureus* remains a significant threat in human and animal populations.

The genetic analysis further reinforces the role of efflux systems in AMR. The targeted genes encode transporters with distinct substrate profiles and biological functions in *S. aureus*: *norA*, *norB*, and *norC* (MFS transporters involved primarily in fluoroquinolone and biocide efflux [32, 35]); *lmrS* (multidrug efflux pump active against macrolides, lincosamides, streptogramins, and antiseptics [16]); msrA (ABC transporter mediating macrolide efflux [36, 37]); and *tet38* (MFS pump associated with tetracycline resistance and host colonization [38, 39]). Beyond antimicrobial efflux, some of these systems influence virulence‐related traits such as biofilm formation and host cell adhesion [35], further increasing their clinical relevance.

The near‐ubiquitous presence of *nor* genes observed in this study is consistent with previous reports [15, 17, 33]. Nevertheless, despite their known association with fluoroquinolone resistance [40, 41], only one isolate exhibited intermediate ciprofloxacin resistance, revealing a genotype–phenotype discrepancy. This pattern extended to other efflux genes. Although *tet38* and *lmrS* were highly prevalent (> 94%), only three isolates were tetracycline‐resistant, and all remained susceptible to lmrS substrates (chloramphenicol, trimethoprim, and linezolid). However, the identification of two genetically distinct (60% similarity) tetracycline‐resistant strains (367 and 369) from a single individual demonstrates co‐colonization and suggests potential for horizontal gene transfer, creating micro‐ecological conditions favorable for local amplification of resistance determinants.

A distinct pattern emerged for *msrA*, the least frequent gene (10.53%) despite high phenotypic erythromycin resistance. Active efflux is the second known mechanism of erythromycin resistance [19, 42], after ribosomal modification [43] and macrolide phosphorylases [18]. These alternative pathways likely explain the high erythromycin resistance in *msrA*‐negative isolates (Figure S1). Nevertheless, the identification of two pairs of genetically identical *msrA*‐positive isolates recovered from different hosts and BHUs provides direct evidence of clonal dissemination of efflux‐associated resistance across host species and geographic boundaries.

Several clones with 100% genetic similarity were recovered from different users within the same and different BHUs (Clusters B and E), from healthcare professionals across units (Cluster C), and from both dogs and humans (Cluster F). This pattern supports the existence of interconnected transmission networks linking hosts and healthcare environments, even in the absence of direct evidence of in situ transmission. In this context, BHUs may function as silent community reservoirs of efflux‐associated AMR, facilitating the circulation of resistance determinants between humans and animals even without overt phenotypic expression. Therefore, the absence of clinically detectable resistance does not necessarily reflect a low epidemiological risk but rather a latent potential for future resistance emergence.

From a One Health perspective, the inclusion of stray dogs proved relevant. Free‐roaming animals were frequently integrated into clusters shared with human isolates, especially in Cluster D, and highly similar canine isolates were identified across different BHUs (Cluster F). Previous studies have highlighted the role of companion and stray animals as reservoirs of clinically relevant *S. aureus* clones [10]. Our findings support this view and indicate that free‐roaming dogs may act as active disseminators within community transmission networks, reinforcing the interconnectedness of human, animal, and environmental health.

Some limitations should be acknowledged. This study was conducted in only three BHUs within a single municipality, providing a localized snapshot of AMR dynamics. Moreover, the PCR‐based approach detects gene presence but does not assess expression levels or functional mutations. Although (GTG)_5_‐PCR proved valuable for initial molecular typing, whole‐genome sequencing would offer higher resolution for confirming strain relatedness and even transmission pathways [20, 21, 25]. Besides, due to limited publications in primary healthcare settings, we necessarily drew comparisons with hospital and ICU literature.

Nevertheless, the detection of *S. aureus* clones harboring MES genes in healthcare professionals, patients, and stray animals highlights their interconnected role in AMR dissemination, supporting a One Health perspective. Under appropriate selective pressure—whether from increased antibiotic use, environmental stressors, or horizontal gene transfer—this latent reservoir may rapidly translate into clinically relevant resistance. These results highlight the importance of sustained surveillance in primary healthcare settings and reinforce the value of proactive, integrated One Health surveillance strategies.

## Author Contributions

The study was conceptualized by Ilderlane da Silva Lopes, Mariana de Barros, Leonardo Moises Sales Bueno, Rodrigo Alves Barros, and Maria Aparecida Scatamburlo Moreira. Data curation was carried out by Ilderlane da Silva Lopes, Ana Júlia Silva Moreira, Mariana de Barros, Jéssica Lobo Albuquerque Caldeira, Leonardo Moises Sales Bueno, and Rodrigo Alves Barros, while formal analysis was performed by Ilderlane da Silva Lopes, Ana Júlia Silva Moreira, Mariana de Barros, Jéssica Lobo Albuquerque Caldeira, Denise Mara Soares Bazzolli, and Maria Aparecida Scatamburlo Moreira. The investigation was conducted by Ilderlane da Silva Lopes, Leonardo Moises Sales Bueno, Ana Júlia Silva Moreira, Rodrigo Alves Barros, and Maria Aparecida Scatamburlo Moreira. All authors contributed to the development, validation, and visualization of the methodology. Software analysis was undertaken by Jéssica Nogueira Rosa, Giarlã Cunha da Silva, Denise Mara Soares Bazzolli, Ana Júlia Silva Moreira, Ilderlane da Silva Lopes, Mariana de Barros, Leonardo Moises Sales Bueno, Rodrigo Alves Barros, and Maria Aparecida Scatamburlo Moreira. Writing the original draft and reviewing and editing the manuscript were carried out by Ilderlane da Silva Lopes, Ana Júlia Silva Moreira, Mariana de Barros, Jéssica Lobo Albuquerque Caldeira, Jéssica Nogueira Rosa, Giarlã Cunha da Silva, Denise Mara Soares Bazzolli, and Maria Aparecida Scatamburlo Moreira. Project administration was led by Rodrigo Alves Barros and Maria Aparecida Scatamburlo Moreira, who also supervised it along with Denise Mara Soares Bazzolli. Maria Aparecida Scatamburlo Moreira provided funding acquisition and resource management.

## Funding

This study was supported by Coordenação de Aperfeiçoamento de Pessoal de Nível Superior—CAPES (Finance Code 001), Conselho Nacional de Desenvolvimento Científico e Tecnológico—CNPq (CNPq/MS‐SCTIE‐Decit no. 01/2018, no. 402562/2018‐6), and Fundação de Amparo à Pesquisa do Estado de Minas Gerais—FAPEMIG. Maria Aparecida Scatamburlo Moreira is a productivity fellow at CNPq.

## Disclosure

All authors read and approved the final manuscript.

## Ethics Statement

The research was approved by the Research Ethics Committee for Human Studies (CEP) under protocol no. 3.014.634 and by the Animal Use Ethics Committee (CEUA) under protocol no. 79/2018, both affiliated with the Universidade Federal de Viçosa. The research was conducted in accordance with the Declaration of Helsinki.

## Consent

Participants signed the informed consent form. The data used in this study were anonymized, and no identifiable information from participants was published.

## Conflicts of Interest

The authors declare no conflicts of interest.

## Supporting Information

Additional supporting information can be found online in the Supporting Information section.

## Supporting information


**Supporting Information** The following supporting information is available with this article: Figure S1: Resistance profile of *Staphylococcus aureus* strains against the tested antimicrobials; Table S1: Primers used for the detection of multidrug efflux system genes in *Staphylococcus aureus* (sequences, product sizes, and references).

## Data Availability

The data are available from the corresponding author upon reasonable request.
