# Supplementary material for: Fingerprinting PCR Reveals Potential Dissemination of Multidrug Efflux System Genes and Antimicrobial Resistance in Staphylococcus aureus Across Primary Healthcare Units in Brazil
Source: Int J Microbiol. 2026 May 14;2026:9287240. doi: 10.1155/ijm/9287240 (PMC13174949; doi:10.1155/ijm/9287240)
Supplement: Supplementary file 1 — Supporting Information The following supporting information is available with this article: Figure S1: Resistance profile of Staphylococcus aureus strains against the tested antimicrobials; Table S1: Primers used for the detection of multidrug efflux system genes in Staphylococcus aureus (sequences, product sizes, and references). [file IJM-2026-9287240-s001.docx]

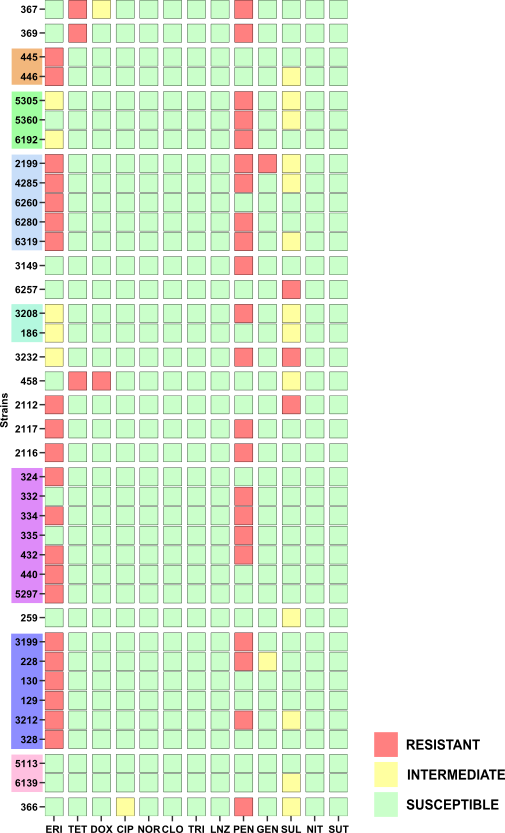


**Figure S1. Resistance profile of *Staphylococcus aureus* strains against the tested antimicrobials.** Isolates are grouped according to fingerprinting similarity clusters Abbreviations: PEN, penicillin; CLO, chloramphenicol; ERI, erythromycin; TRI, trimethoprim; DOX, doxycycline; TET, tetracycline; GEN, gentamicin; NOR, norfloxacin; SUL, sulfonamide; NIT, nitrofurantoin; SUT, sulfamethoxazole-trimethoprim; CIP, ciprofloxacin; LNZ, linezolid.

**Table S1. Primers used for the detection of Multidrug Efflux System genes in *Staphylococcus aureus.***

| **Gene** | **Sequence 5’-3’** | **Product (bp)** | **Antibiotic Resistance** | **Reference** |
| --- | --- | --- | --- | --- |
| *norA* | *f* - TTCACCAAGCCATCAAAAAG  *r* - CTTGCCTTTCTCCAGCAATA | 620 | Hydrophilic fluoroquinolones (e.g., ciprofloxacin, norfloxacin) | Pourmand, et al. (2014); Sinha et al. (2024) |
| *norB* | *f* - AGCGCGTTGTCTATCTTTCC  *r* - GCAGGTGGTCTTGCTGATAA | 213 |  | Hassanzadeh et al. (2017); Sinha et al., (2024) |
| *norC* | *f* - AATGGGTTCTAAGCGACCAA  *r* - ATACCTGAAGCAACGCCAAC | 216 |  |  |
| *msrA* | *f* - TCCAATCATTGCACAAAATC  *r* - AATTCCCTCTATTTGGTGGT | 163 | Macrolides (e.g., erythromycin), lincosamides (e.g., lincomycin, clindamycin), | Martineau et al. (2000); Sinha et al. (2024) |
| *lmrS* | *f* - GCAAGCTTATGGCTAAAGTTGAATTAACAAC  *r* - GCGGATCCTTAAAATTTCCTTCTATTACTTT | 1400 | Multidrug resistance: linezolid, chloramphenicol, florfenicol, trimethoprim, erythromycin, kanamycin, lincomycin, streptomycin | Floyd et al. (2010); Sinha et al. (2024) |
| *tet38* | *f* - TTCAGTTTGGTTATAGACAA  *r* - CGTAGAAATAAATCCACCTG | 400 | Tetracyclines (e.g., tetracycline, oxytetracycline); | Truong-Bolduc et al. (2006); Sinha et al. (2024) |
